# Digital Subtraction Angiography Contrast Material Transport as a Direct Assessment for Blood Perfusion of Middle Cerebral Artery Stenosis

**DOI:** 10.3389/fphys.2021.716173

**Published:** 2021-08-05

**Authors:** Yun-Hao Lu, Yan Cai, Yi Zhang, Rui Wang, Zhi-Yong Li

**Affiliations:** ^1^School of Biological Sciences and Medical Engineering, Southeast University, Nanjing, China; ^2^Center of Interventional Radiology and Vascular Surgery, Department of Radiology, Zhongda Hospital, Medical School, Southeast University, Nanjing, China; ^3^School of Mechanical, Medical and Process Engineering, Queensland University of Technology, Brisbane, QLD, Australia

**Keywords:** contrast material perfusion, middle cerebral artery, stenosis, computational fluid dynamics, interventional surgery, DSA

## Abstract

Digital subtraction angiography (DSA) is a fluoroscopic technique used extensively in interventional radiology for visualizing blood vessels. It has also been used to evaluate blood perfusion. However, the perfusion obtained in previous techniques was extracted from signal intensity rather than by the transport of contrast material (CM) through blood flow. The main aim of this study is to evaluate the morphological effects on the hemodynamics and the CM concentration in the middle cerebral artery (MCA) stenosis. We proposed a quantitative parameter, i.e., contrast material remaining time (CMRT), to describe the variation in the transport of CM over time. Computational fluid dynamics simulations were performed on both reconstructive synthetic and patient-derived models. In the synthetic models, we evaluated the variation of flow patterns and the transport of CM with different degrees of stenosis and the location of the lesion. It was found that an increase in the degree of stenosis (from 30 to 80%) resulted in a significant increase in CMRT at the anterior cerebral artery (ACA) outlet (*p* = 0.0238) and a significant decrease in CMRT at the MCA outlet (*p* = 0.012). The patient-derived models were reconstructed from the pre- and post-interventional DSA images of a patient with MCA stenosis. Both blood flow velocity and CMRT increased at the ACA outlet but decreased at the MCA outlet. The perfusion analysis demonstrated that the perfusion function was improved after interventional surgery. In conclusion, changes in stenotic degree at MCA may lead to apparent differences in the hemodynamic distribution and the transport of CM. CMRT could be a quantitative indicator to evaluate the changes in blood perfusion after the intervention for MCA stenosis.

## Introduction

In recent years, the increase in morbidity and mortality from cardiovascular disease (CAD) leads to a significant increase in atherosclerosis diagnoses and interventional surgeries (Ahmed et al., 2012; Nevidomskyte et al., 2019; Zhao et al., 2019), whereas the noninvasive imaging techniques, such as CT and MRI, provide the objective data with the prognostic value. However, the digital subtraction angiography (DSA) is still the “golden standard” for evaluating artery anatomy, especially at the stenotic lesion (Chng et al., 2008; Ma et al., 2010; Chien and Viñuela, 2013; Ionita et al., 2014; Akiyama et al., 2016; Huang et al., 2016). The ascendancy of DSA is that the vascular lesions may have real-time image feedback with iodine-based contrast material (CM). As such, DSA is widely used to monitor and guide minimally invasive endovascular interventions (Chng et al., 2008; Ionita et al., 2014; Cheon, 2015). However, the visual assessment of flow is dependent on the judgment of the operator according to the DSA image series. Therefore, there is a need for quantitative or parametric descriptions of blood flow through the vasculature by extracting the temporal information of the DSA, which would add its value to the ability to both diagnose and determine the effects of therapeutic interventions (Divani et al., 2006; Strother et al., 2010).

There have been several techniques to investigate the functional information on blood perfusion based on the DSA images. For example, a two-dimensional (2D) perfusion imaging can detect an increased pixel density when CM flows through a specified region of interest (ROI) (Simonsen et al., 1999; Divani et al., 2006; Cheon, 2015; Khan et al., 2017). Meanwhile, the perfusion parameters, such as arrival time (AT) and time to peak (TTP) signal density, can be assessed from the time–density curve (TDC). This technique has been previously used in the analysis of perfusion changes in intracranial vascular disease (i.e., carotid stenosis, carotid-cavernous fistula, and moyamoya disease) (Zaharchuk, 2011; Cheon, 2015; Akiyama et al., 2016; Leng et al., 2019), arterial stenosis of the lower extremity, and the pretherapeutic assessment during cerebrovascular interventional surgery (Meckel et al., 2007; Hiramoto et al., 2018). However, the aforementioned perfusion parameters are extracted from the changes in signal intensity rather than the transport of CM through the blood flow. The mechanisms of how the flow affects CM diffusion and convection are yet to be explored. Importantly, such an understanding is necessary to evaluate the improvement in blood perfusion for cerebral vascular diseases based on the pre- and post-interventional DSA images.

In this study, we hypothesized that the transport of CM through the artery is associated with stenotic morphology. In this study, we focused on the stenosis of the middle cerebral artery (MCA). The simulations of computational fluid dynamics (CFD) at the MCA stenosis and the transport of CM were first performed on the synthetic models with stenotic level and location variants. It was found that the flow patterns, such as flow velocity and pressure, would be influenced by artery morphology and, therefore, would affect the distribution of CM concentration. Then, the perfusion analysis was performed on the patient-derived models based on the pre- and post-interventional DSA images of a patient with MCA stenosis. We proposed a quantitative evaluation, i.e., contrast material remaining time (CMRT), to describe the variation in the transport of CM. The correlation between CMRT and stenotic morphology was investigated on synthetic models. In addition, the changes in CMRT before and after intervention were compared with the perfusion parameters obtained from the DSA series.

## Materials and Methods

### Computational Fluid Dynamics Modeling

#### Synthetic Model

**Figure 1:**
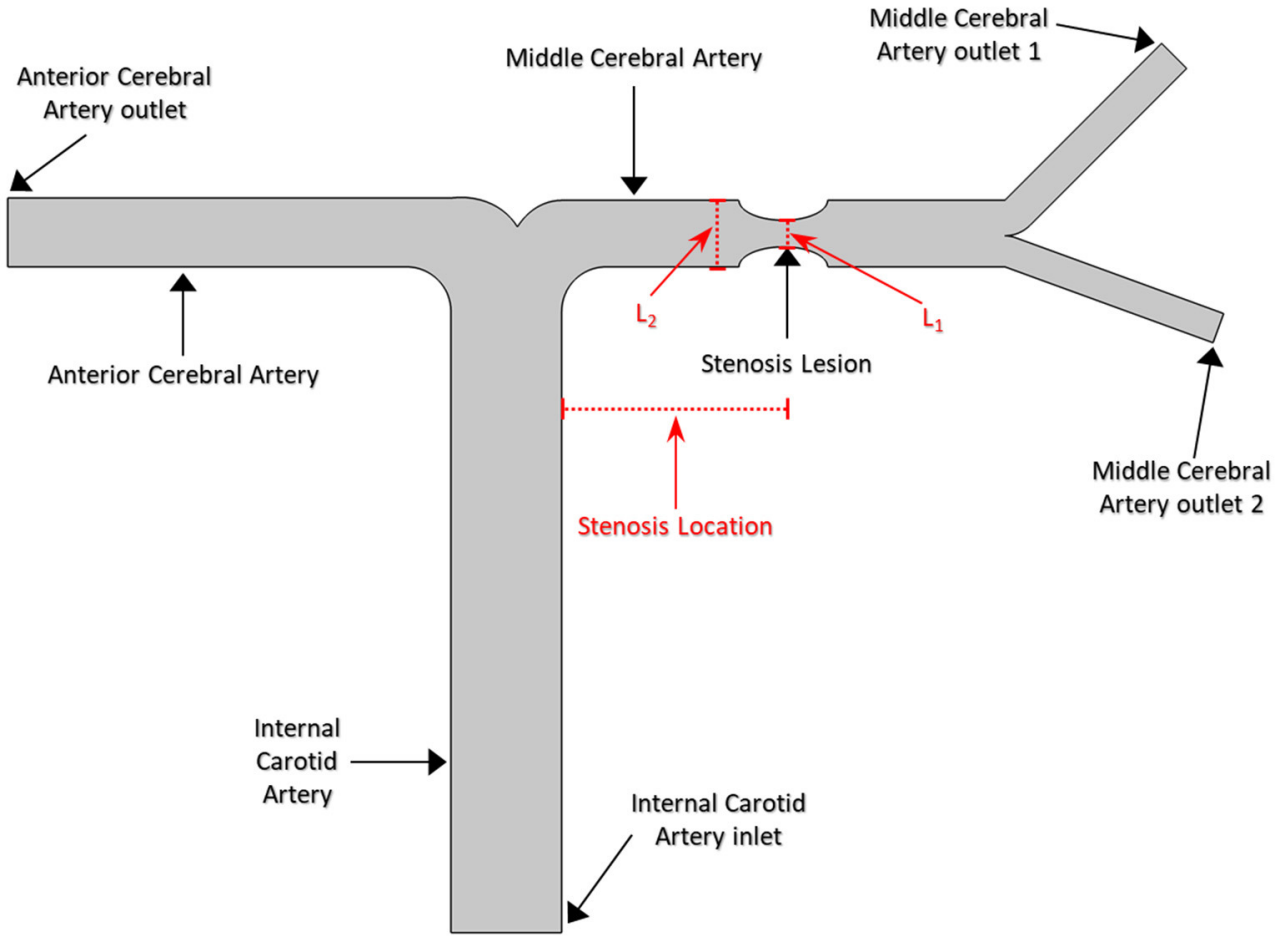
The geometry of the middle cerebral artery and bifurcation in the model.

A series of 2D synthetic models of MCA stenosis were constructed using COMSOL Multiphysics^®^ (COMSOL Inc., Stockholm, Sweden). As shown in Figure 1, the baseline model consisted of a vertical internal carotid artery (ICA) of 30 mm, followed by a bifurcation of a horizontal anterior cerebral artery (ACA) of 20 mm and a horizontal MCA of 20 mm. A 60% stenosis was located at the middle of MCA, i.e., the center position of the stenotic lesion was 10 mm away from the bifurcation. Additional models were established by varying the locations of the stenotic lesion and the stenotic levels (indicated by the length L1 divided by the length L2 in Figure 1). We created a total of nine different synthetic models with the stenotic levels of 30, 60, and 80% and the lesion locations of anterior (5 mm away from the bifurcation), middle (10 mm away from the bifurcation), and posterior (15 mm away from the bifurcation) of MCA. The MCA was further bifurcated into two arterioles with an angle of 75° (Jones, 2014). The inlet of the model was located at ICA, while the outlets included one at ACA and two at MCA. Table 1 listed the diameter and length of the arteries in the models.

**Table 1:** The diameter and length for cerebral artery system.

**Arteries diameter/length**	**Size**	**References**
Anterior cerebral artery diameter	3.1 mm	Stefani et al. (2000) and Nevidomskyte et al. (2019)
Anterior cerebral artery length	20 mm	Aggarwal et al. (2012)
Middle cerebral artery diameter	3 mm	Serrador et al. (2000)
Middle cerebral artery length	20 mm	Umansky et al. (1985)
Bifurcation of middle cerebral artery diameter (top/bottom)	1.6 mm/ 1.4 mm	Rai et al. (2013)
Bifurcation of middle cerebral artery length	10 mm	Rohan et al. (2014)
Internal carotid artery diameter	5 mm	Khan et al. (2017)
Internal carotid artery length	30 mm	Choudhry et al. (2016)

#### Patient-Derived Model

**Figure 2:**
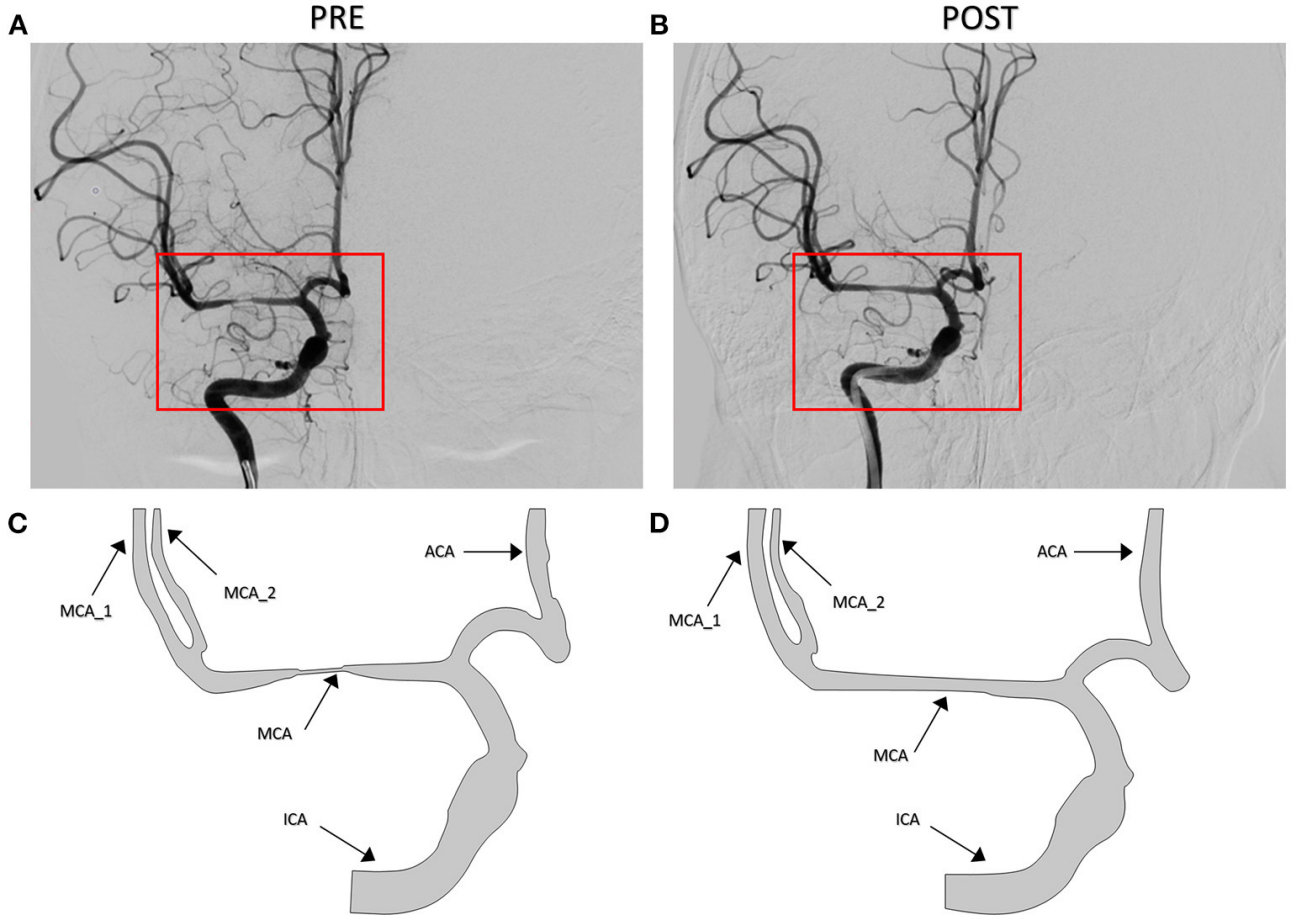
Digital subtraction angiography (DSA) images of the patient **(A,B)** and patient-derived model **(C,D)**. **(A,C)** Pre-interventional, labeled as PRE. **(B,D)** Post-interventional, labeled as POST.

The patient-derived models were reconstructed based on the pre- and post-interventional DSA images at the arterial phase of a patient with MCA stenosis (Figure 2). We focused on the bifurcation region from ICA into ACA and MCA (which is shown as the red rectangular window in the figure). The pre- and post-interventional 2D models with MCA stenosis were constructed according to the morphological information obtained from DSA images. We labeled these two patient-derived models as PRE and POST, respectively. Subsequently, the CFD analysis was performed on these two models to investigate the hemodynamics in the arteries and mass transport of CM.

#### Mass Transport Equations

The steady-state laminar flow was performed on both synthetic and patient-derived models, which was governed by the Navier–Stokes equations as follows:

(1) $$\rho [\frac{\partial u}{\partial t}+\left(u\;\cdot \;\nabla \right)\;u]=-\nabla p+\mu \nabla^{2}u+\;f$$

It was assumed that the CM injected at the ICA was thoroughly mixed with the blood flow instantly. Then, the transport of the CM followed the convection–diffusion equation as follows:

(2) $$\begin{array}{l} \frac{\partial c}{\partial t}+u\nabla \cdot c=\nabla \cdot \left(D\nabla c\right)+\Phi \end{array}$$

where *c* is the concentration of CM; ***u*** is the blood flow velocity; *D* = 2.5 × 10^−8^ *m*^2^/*s* is the diffusion coefficient of CM in the blood (Jost et al., 2011); and Φ is the source term caused by the injection of CM; in this study, Φ is set as 0.5 *mol*/*m*^3^.

#### Computational Fluid Dynamics Analysis

The meshing procedure was performed in COMSOL Multiphysics version 5.3 to generate a computational grid with a physics-controlled mesh of a total of 4,744 elements. The mesh-independent analysis was implemented to ensure that enough grids were constructed to capture the variations of CFD parameters.

**Figure 3:**
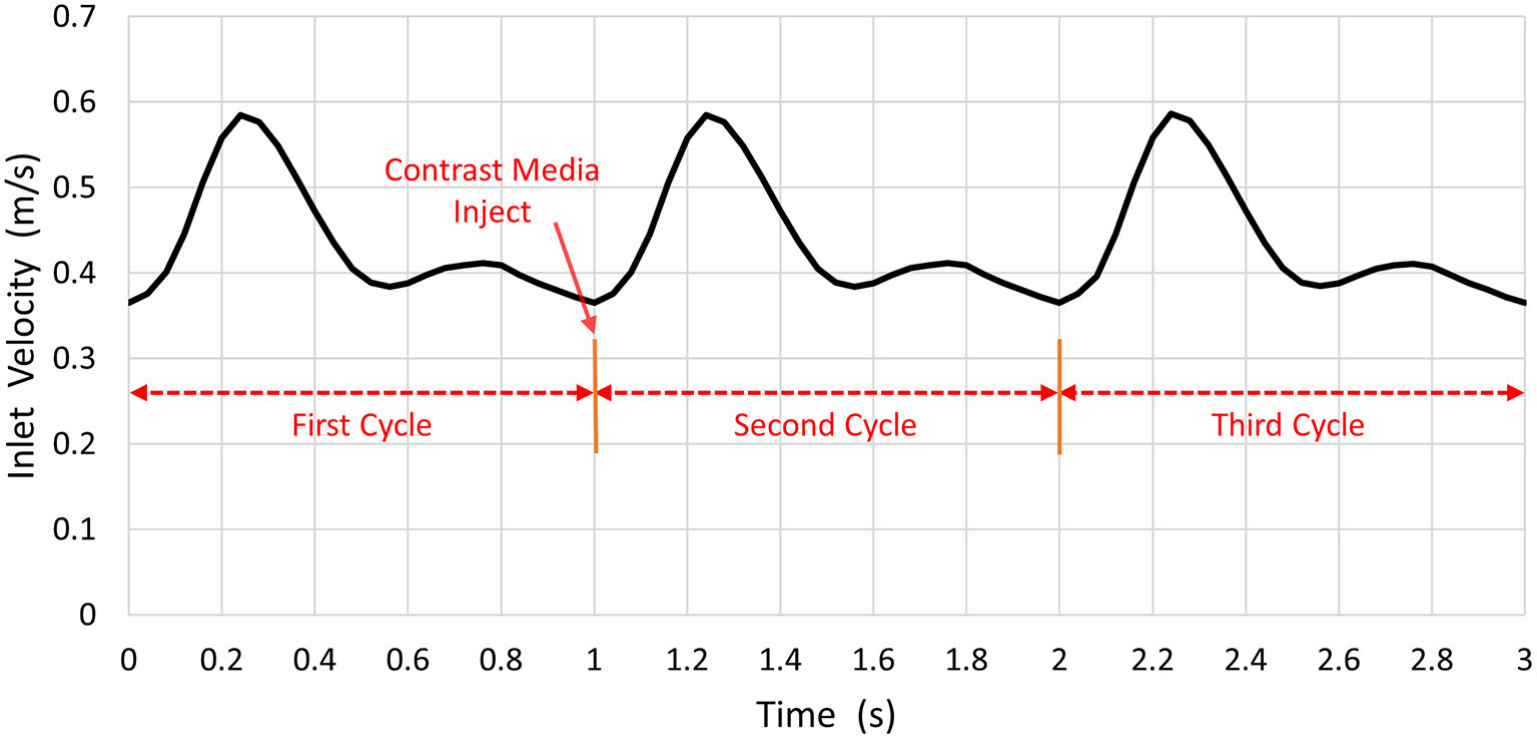
The blood inlet velocity.

The inlet pulsatile flow rate was derived from the phase-contrast MR imaging data used for synthetic and patient-derived models (Figure 3). The pressures at the ACA and MCA outlets were set to 0 Pa (Bowker et al., 2010; Lauric et al., 2014). Blood was assumed to be an incompressible Newtonian fluid with a density of 1, 060 *kg*/*m*^3^ and a viscosity of 3.5 × 10^−3^ *Pa* • *s* in the simulation (Palareti et al., 2016; Leng et al., 2019). The arterial wall was assumed to be rigid without displacement.

The transient CFD simulation was performed with COMSOL Multiphysics 5.3 for three cardiac cycles. Three cycles (i.e., 1 s per cycle) were simulated. At the start point of the second cardiac cycle, the CM was injected from the ICA inlet. The time step was 0.0045 s, and the convergence criterion was satisfied when the residual of continuity was <10^−4^.

### Perfusion Analysis Based on DSA Images

Digital subtraction angiography acquisitions with a standard, clinically routine protocol for the patient with MCA stenosis were performed at pre- and post-intervention. The DSA acquisition was performed using a biplane angiography suite (Artis-One; Siemens, Erlangen, Germany) with a standard, clinically routine protocol. Both pre- and post-interventional angiograms were obtained by using the same catheter in the same position. The degree of MCA stenosis was measured according to the North American Symptomatic Carotid Endarterectomy Trial (NASCET) criteria by an experienced neuroradiologist (Doyle et al., 2014). A bolus of 8 mL of diluted CM (i.e., 320 mg I/mL) was injected into patients at an injection rate of 5 mL/s by contrast delivery system (Angiomat Illumena; Liebel-Flarsheim, Cincinnati, OH, USA).

**Figure 4:**
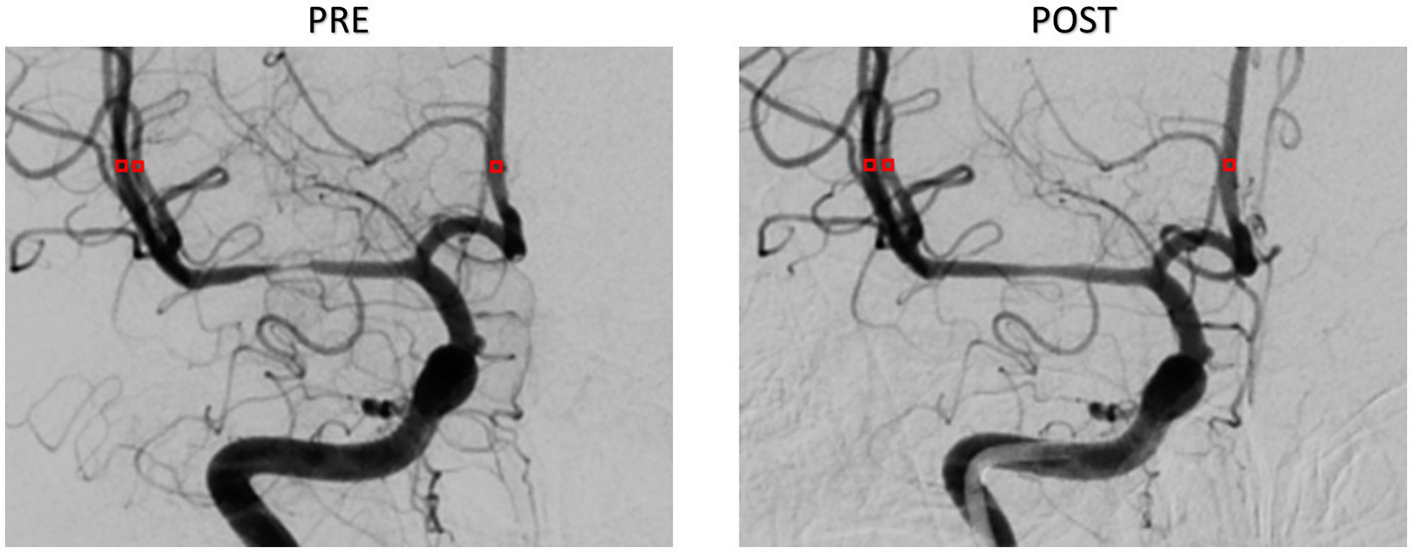
Region of interest location selection on DSA images.

A series of DSA images were extracted and analyzed in MATLAB^®^ software (MathWorks Inc., Natick, Massachusetts, USA). The size of the DSA image was 512 × 512 pixels, and the gray-scale value ranged from 0 to 255. The shooting frequency of DSA sequences was four frames per second (fps). Each series of DSA images contained two phases, namely, the arterial phase and the capillary phase, which involved from the beginning of the injection of CM until the clearance of CM through the capillaries. Then, we selected three ROIs of 3 × 3 pixels from object vessel MCA and ACA in DSA, which are shown as red squares in Figure 4. The TDC was generated from the average pixel gray-scale value within the ROI. Finally, the third-order Hermite polynomial interpolation was conducted to smooth the TDC.

The two functional parameters, namely, TTP and mean transit time (MTT), were calculated to quantify the perfusion status. TTP represents the time from the start of the TDC to the appearance of the highest point, which could estimate the blood flow velocity. MTT presents the time to half-peak of the integration curve for CM, which was used to estimate the blood flow velocity and volume.

### Contrast Material Remaining Time

To quantitatively evaluate the CM passing through the arteries, we proposed CMRT as a functional parameter in his model. CMRT was defined as the difference between the two time points t1 and t2, where t1 was the time when the concentration increased to reach 99% of the peak value, and t2 was the time when the concentration decreased to 1% of the peak value (see solid red line in **Figure 7**). Obviously, CMRT indicated the transportation of CM through the arteries and was associated with hemodynamics within the arteries.

### Statistical Analysis

The Spearman's correlation analysis was applied in this study to verify the correlation between the transport of CM and the vessel morphology. The ranks of the Spearman's correlation coefficient were calculated between CMRT and stenotic levels, as well as CMRT and stenotic locations separately. Statistical analyses were performed using GraphPad Prism 8.0 software (GraphPad Software Inc., Northside, San Diego, USA). *P* < 0.05 was considered statistically significant.

## Results

### Synthetic Models

#### Hemodynamic Results

**Figure 5:**
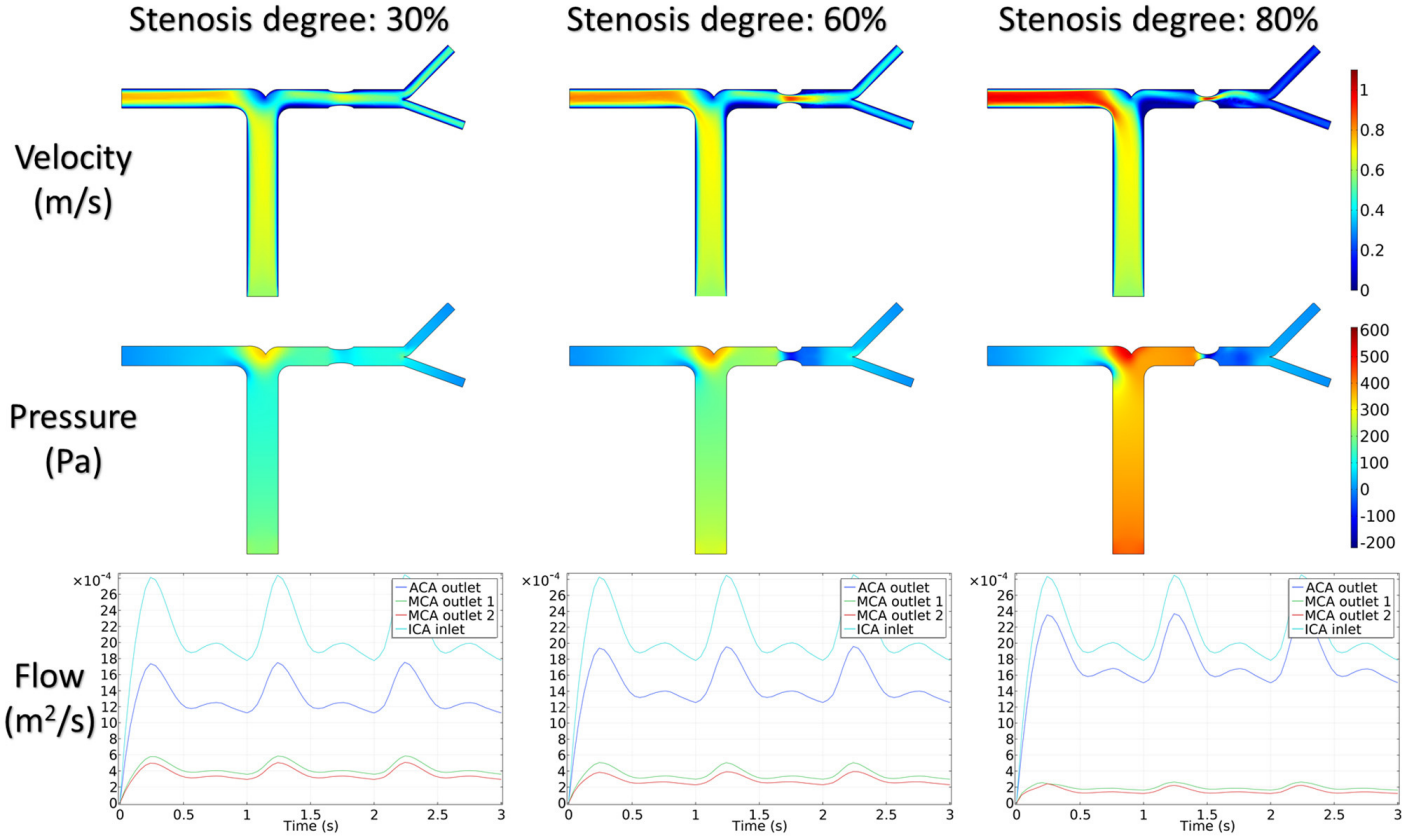
Velocity, pressure, and inlet/outlet flow alternation with the degree of stenosis.

**Figure 6:**
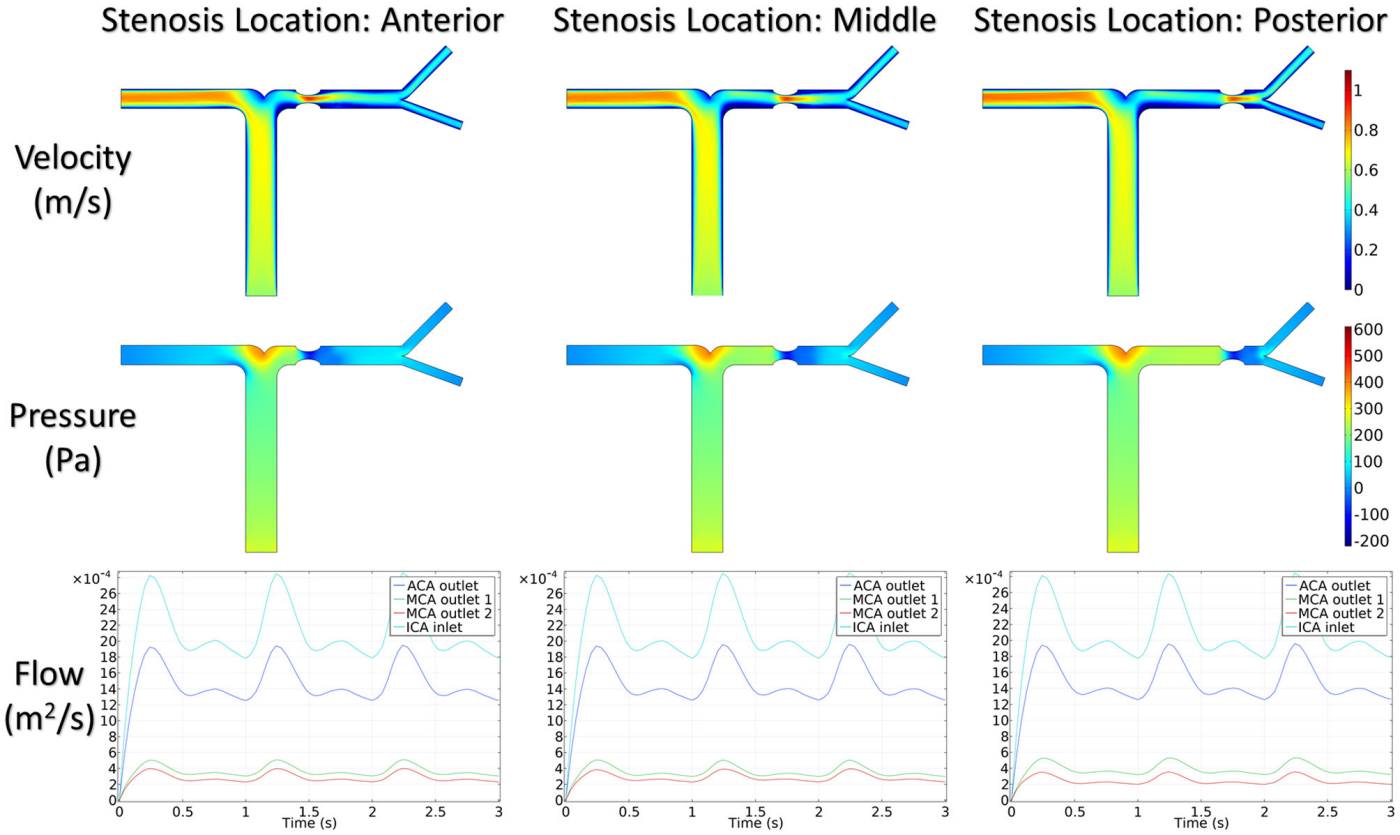
Velocity, pressure, and inlet/outlet flow alternation with the location of stenosis.

Figure 5 shows the distribution of hemodynamic results, such as velocity, pressure, and inlet/outlet flow at the systole, on synthetic models with different stenotic levels. A sharp increase in velocity at the stenotic lesion and a decrease in downstream were observed in all synthetic models. Specifically, the decrease in velocity and pressure along the stenotic lesion was highly influenced by the stenotic levels. In severe stenosis (i.e., stenotic level = 80%), the distal velocity at the MCA reduced to 0.2 m/s compared with the mild case (i.e., stenotic level = 30%) with 0.5 m/s. In addition, the pressure drop at the stenotic lesion increased from 100 to 450 Pa when the degree of stenosis increased. Figure 6 shows the distribution of hemodynamic results on synthetic models with different stenotic locations. Minor differences can be found in these synthetic models, suggesting that the effects of stenotic location on velocity and pressure distributions are negligible.

The stenotic level further impacted the outlet flow at MCA and ACA remarkably. As shown in Figure 5, the outlet flow at ACA increased with the stenotic level at the MCA due to the flow conservation. Consequently, the significant reduction in outlet flow at MCA suggested a low level of perfusion through the stenotic MCA. Compared with the stenotic level, the stenotic location has less influence on the ACA and MCA outlet flow (Figure 6). However, a tremendous difference in the outlet flow at two MCA outlets was detected in the case of the posterior stenotic location.

#### Transportation of CM

**Figure 7:**
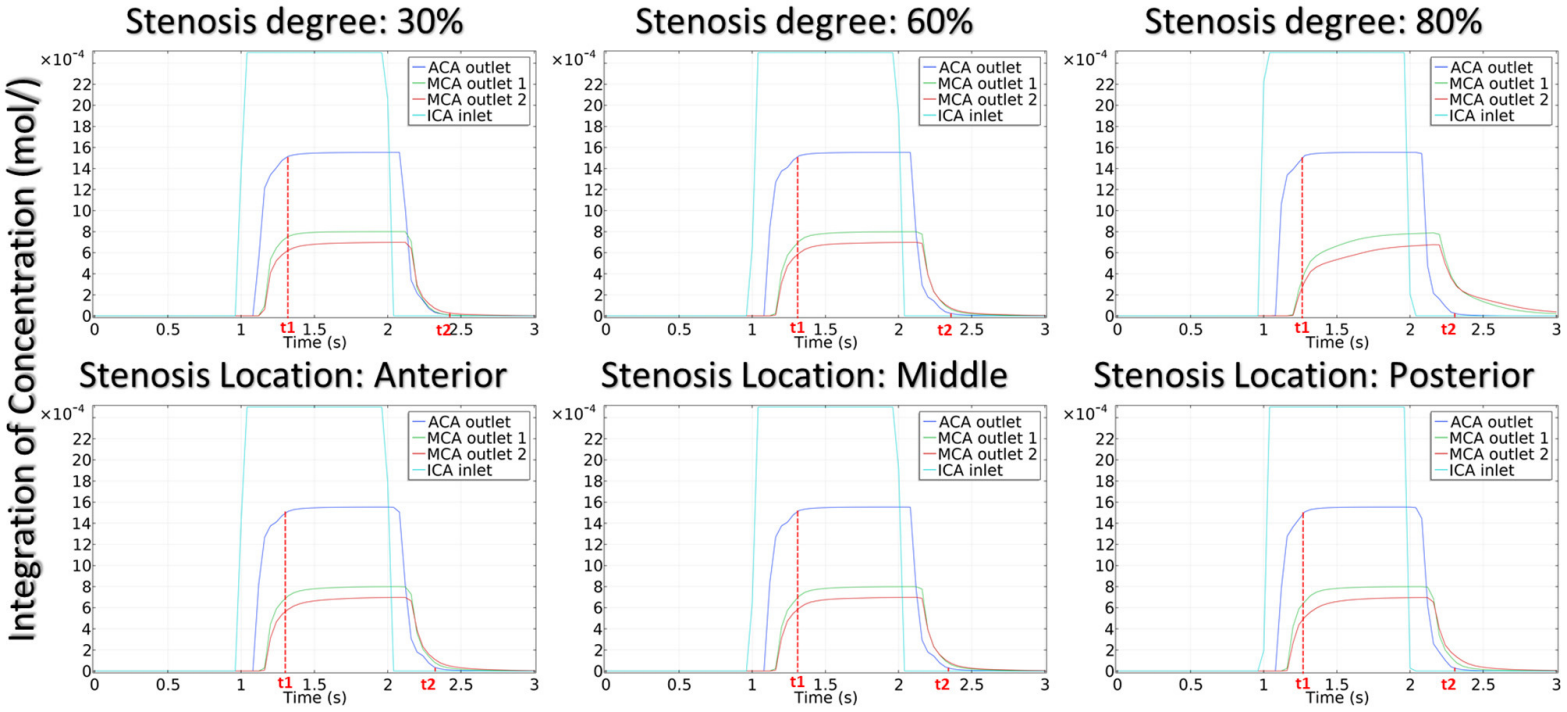
The integration of contrast material concentration at inlet and outlets.

The integration of CM concentration at the ICA inlet and other three outlets is shown in Figure 7. Similar to the effects of stenotic level on hemodynamics, the transport of CM was significantly influenced by the stenotic level rather than the stenotic location. It is noteworthy that the time for CM to reach the peak value at the MCA outlets was delayed significantly in the severe stenotic case.

To assess the delay effect of the transport of CM caused by different stenotic morphologies, the CMRT at three outlets was calculated in all synthetic models and compared in Table 2. With an increase in the stenotic level, the CMRT in ACA decreased slightly, while a sharp increase in CMRT was observed in both MCA outlets. This result indicated that the delay effect of the transport of CM was exacerbated by the stenotic level since the convection of CM was related to the hemodynamics in the stenotic artery. On the contrary, there were slight differences in CMRT among models with varied stenotic locations.

**Table 2:** The contrast material remaining time (CMRT) for the synthetic model.

**Model**	**ACA CMRT (s)**	**MCA-1 CMRT (s)**	**MCA-2 CMRT (s)**
Anterior 30%	1.08	1.12	1.28
Anterior 60%	1.04	1.24	1.36
Anterior 80%	1.04	1.48	1.64
Middle 30%	1.08	1.08	1.28
Middle 60%	1.04	1.2	1.36
Middle 80%	1.04	1.6	1.8
Posterior 30%	1.08	1.08	1.32
Posterior 60%	1.04	1.2	1.4
Posterior 80%	1.04	1.64	1.68

The correlation between CMRT and stenotic locations as well as CMRT and stenotic degrees was analyzed using the Spearman's correlation method. Table 3 illustrates that there was no significant correlation between CMRT and stenotic locations (*P*-values were 0.99, 0.90, and 0.58). However, significant correlations between CMRT and stenotic degree were demonstrated. It is found that the CMRT of the ACA outlet had a negative correlation with the stenotic level (*r* = −0.866, *P* = 0.0238), and the CMRT of the MCA outlets had a positive correlation with the stenotic level (*r* = 0.9567, *P* = 0.0012).

**Table 3:** The correlation between CMRT and morphology.

**Effect variable**	**Artery outlet**	***r***	***P***
Stenotic location	ACA CMRT	0	>0.9999
	MCA-1 CMRT	−0.05315	0.9095
	MCA-2 CMRT	0.2126	0.5869
Stenotic degree	ACA CMRT	−0.866	0.0238^*^
	MCA-1 CMRT	0.9567	0.0012^*^
	MCA-2 CMRT	0.9567	0.0012^*^

* *represented statistically significant*.

### Patient-Derived Model

#### Velocity and Pressure

**Figure 8:**
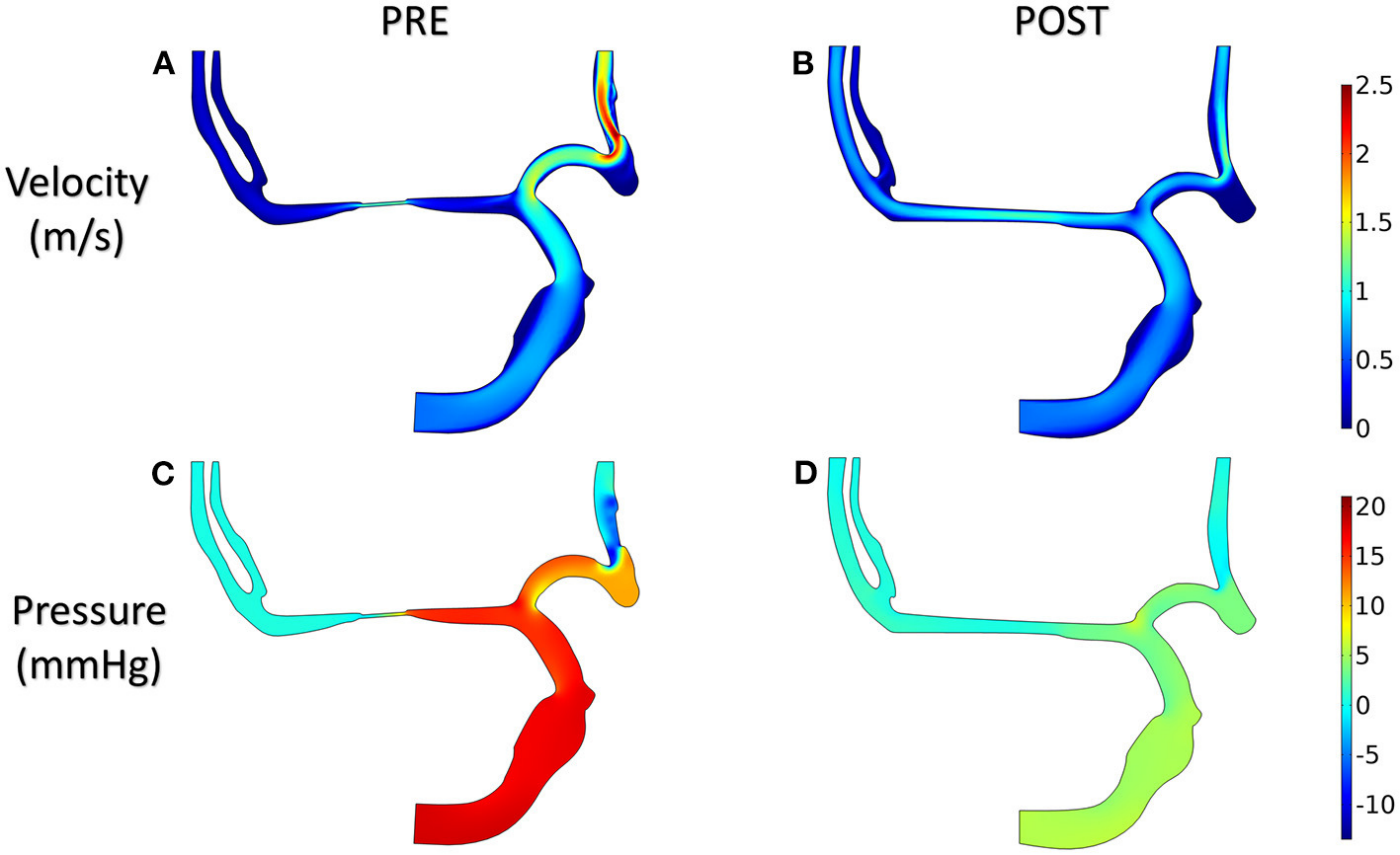
Velocity **(A,B)** and pressure **(C,D)** in the patient-derived model.

The distributions of blood velocity and pressure for the patient-derived models are shown in Figure 8. Due to the severe stenosis at MCA, the compensatory effect of ACA can be found explicitly from the velocity field, which reached up to around 2 m/s at ACA in the PRE model. After interventional surgery, the stenotic degree was decreased from 83 to 20%, while the blood perfusion through MCA was improved significantly. At the two MCA outlets, the velocity increased from 0.3 to 0.8 m/s. Furthermore, in the PRE model, the higher pressure occurred at ICA and part of ACA, and the pressure gradient reached 10 mm Hg at the stenotic lesion. In contrast, the distribution of pressure became more homogeneous in the POST model, and the pressure drop at the stenotic lesion was decreased to 4 mm Hg.

#### Concentration of Contrast Material

**Figure 9:**
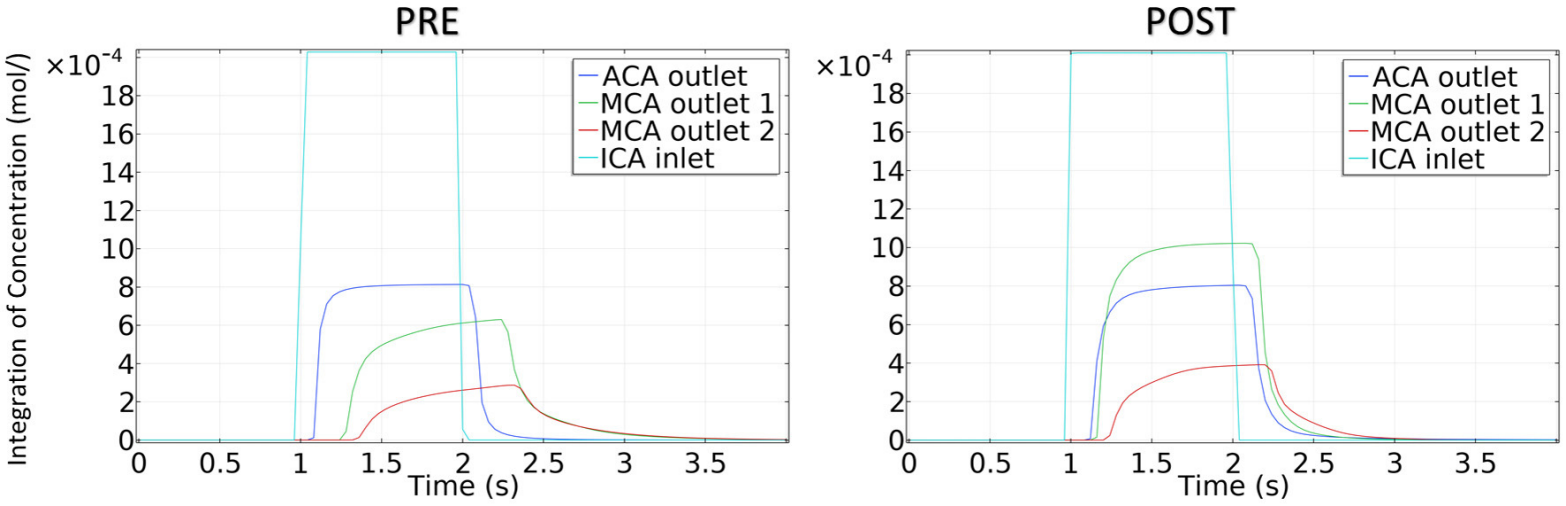
The concentration of contrast material in the patient-derived model.

The concentration of CM in the patient-derived model is shown in Figure 9. Following interventional surgery, there was a remarkable increase in the CM concentration through the MCA, indicating the improved blood perfusion in the stenotic MCA due to the morphological change. In addition, the TTP value at both MCA outlets in the POST model decreased compared with the PRE model, which also provided evidence for the improved blood perfusion in MCA by the surgery.

#### Contrast Material Remaining Time

To quantitatively compare the transport of CM in the patient-derived model, we calculated the CMRT, as defined in synthetic models. The results shown in Table 4 revealed the changes in CMRT in the ACA and MCA outlets before and after the intervention. The CMRT increased after surgery from 1.24 to 1.64 s in the ACA outlet and decreased from 1.76 to 1.28 s in MCA outlet 1 (MCA-1) and from 1.84 to 1.44 s in MCA outlet 2 (MCA-2). Both the increase in CMRT in ACA and the decrease in CMRT in MCA demonstrated the effect of interventional surgery on the transport of CM.

**Table 4:** The CMRT for the patient-derived model.

**Model**	**ACA CMRT(s)**	**MCA-1 CMRT(s)**	**MCA-2 CMRT(s)**
PRE	1.24	1.76	1.84
POST	1.64	1.28	1.44

### Perfusion Analysis

**Figure 10:**
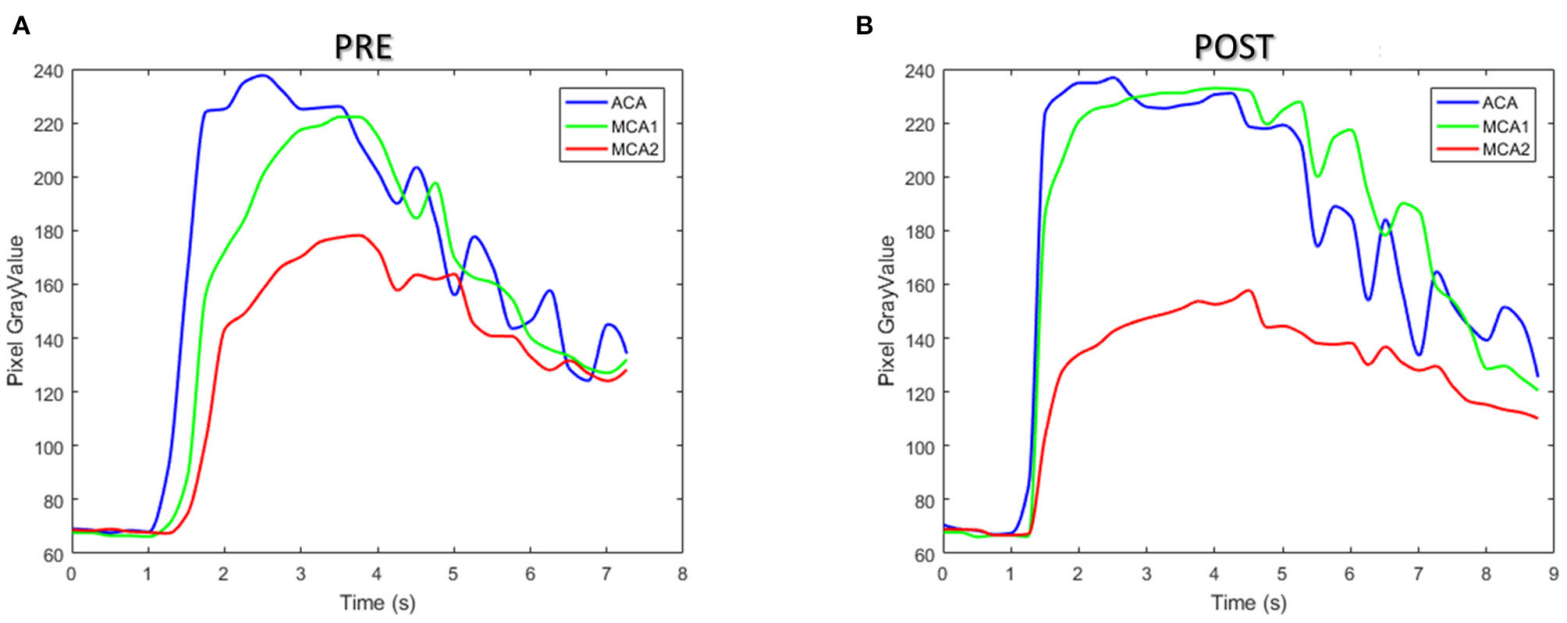
The time–density curve of the patient between pre- **(A)** and post-interventional **(B)** surgery.

Figure 10 illustrates the TDCs of the patient in ROIs defined at ACA and MCA-1 and MCA-2 before (Figure 10A) and after intervention (Figure 10B). A significant increase in CM concentration in MCA-1 can be found in the TDC of the post-intervention case. Accordingly, the perfusion parameters (i.e., TTP and MTT) were obtained from the TDCs, as summarized in Table 5. It was found that both the TTP and the MTT increased in the two MCAs.

**Table 5:** The perfusion parameters at pre- and post-intervention.

	**TTP (s)**			**MTT (s)**		
**Model**	**ACA**	**MCA-1**	**MCA-2**	**ACA**	**MCA-1**	**MCA-2**
PRE	2.51	3.51	3.76	3.54	3.73	3.84
POST	2.51	4.01	4.51	4.2	4.35	4.45

## Discussion

### Morphology Impacts Flow Patterns

In this study, we demonstrated that the alternation of stenotic morphology affects the transport of CM due to the change in the flow pattern on both synthetic and patient-derived models. In the synthetic model, both the stenotic level and location played essential roles in the variation of hemodynamics in MCA. It is noteworthy that a higher stenotic degree induced a significant difference in the flow pattern between ACA and MCA segments (Wu et al., 2018). In addition, the patient-derived model also demonstrated that the local blood perfusion at the stenotic lesion was improved after interventional surgery due to the enlarged MCA. Furthermore, both synthetic and patient-derived models demonstrated the opposite variations in the velocity of both ACA and MCA segments, i.e., when one goes up, the other goes down, and vice versa. Being another crucial hemodynamics index, the pressure of stenotic cerebral arteries also shows the remarkable impact induced by the alternative vessel morphology. The translesional pressure of pre- and post-stenosis obviously increases with the degree of stenosis in the synthetic model. Besides, the translesional pressure distinctly declines after interventional surgery. This result is consistent with the clinical observation obtained by the insertion of an angiocatheter (Poon et al., 2015).

### Contrast Material Remaining Time in Synthetic and Patient-Derived Models

In this study, we proposed that the CMRT, which represented the CM residence time in cerebral vessels, was a quantitative parameter. Compared with the perfusion analysis based on the pixel density of DSA images, the CMRT was calculated directly from the transport of CM through the stenotic artery. The results of CMRT showed that both synthetic and patient-derived models have a similar variation tendency of the transport of CM and diffusion in ACA and MCA segments, especially in the pre-interventional patient-derived model. In this model, the CMRT result was approximately similar to that of the 80% degree of stenosis in the synthetic model. The results also showed that the CMRT was strongly correlated with the stenotic level. In particular, the CMRT in the ACA segment was negatively correlated with the stenotic level and the CMRT in the MCA segment was positively correlated with the stenotic level. These results demonstrated that CMRT provided helpful information for cerebral artery hemodynamics and CM perfusion status through the stenotic lesion. However, two basic principles should be followed before applying CMRT in clinics. First, the CM injection catheter should be placed as far away as possible from the stenotic lesion to ensure that CM is entirely mixed with blood (Gibson and Bodenham, 2013). Second, to obtain the proper spatial morphology of blood vessels, DSA images should be taken from different angles to avoid disturbing and overlapping from the X-ray shooting direction.

### Perfusion of Patient-Derived Model

The perfusion information of the patient was extracted from TDC and was analyzed with two parameters, namely, TTP and MTT. The TDC of the ROIs in PRE and POST models showed a similar tendency due to the alternation in CM concentration in the patient-derived model. In particular, both TDC and CM concentrations in the MCA-1 rose after surgery, and their values became higher than that in the ACA segment. Although both TTP and MTT increased in the two MCA outlets after surgery, which is inconsistent with other reports of the DSA analysis of cerebral perfusion, it is noteworthy that the TTP value is increased remarkably in the POST model, suggesting that the CM perfusion is improved by the surgery (see green lines in Figure 10).

### Study Limitations and Future Work

First, to verify the reliability and clinical application of the CMRT, extensive clinical studies are needed to evaluate the relationship of CMRT with more morphological and hemodynamic parameters. In this study, we demonstrated the correlation of CMRT with stenotic morphology using synthetic models. While in the validation of patient cases, one patient may not be enough to demonstrate its clinical significance. Future large-scale follow-up patient studies are needed to establish the relationship between CMRT and changes in perfusion status in different patient conditions. Furthermore, extensive validation studies are also needed to translate our method of using CMRT as a real-time monitoring tool for interventional surgery.

Second, the hemodynamic and transportation model was a 2D model. The geometry of the arteries in the patient-derived model was reconstructed from DSA projection images, in which the overlapped blood vessels can be hardly distinguished from the image. With more available three-dimensional (3D) information of vessels, the 3D patient-derived models can be constructed to assess more accurate alteration in morphology and hemodynamics. However, the benefit of the 2D model is that it can significantly reduce the computational time for CFD simulation, making it highly feasible in clinical practice.

Third, the capillary and venous phase was not included in the perfusion analysis of TDC. In addition, since the frequency of the X-ray image taken was 4 fps, the time interval in each discrete image was 0.25 s. Therefore, the TDC was not precise enough to capture each moment when blood flows through the stenotic artery. To improve the accuracy, the frequency of the image taken could be increased to monitor more precise blood flow status.

## Conclusion

To describe the variation of the transport of CM with time, we proposed a quantitative parameter “CMRT” that was obtained from the transport calculation of CM. In both synthetic and patient-derived models, a higher stenotic level leads to a higher flow rate and a higher CM concentration in ACA, but a lower flow rate and a lower CM concentration in MCA. Both synthetic and patient-derived models demonstrated the significant correlation of CMRT with the morphological variation. In addition, the changes in CMRT in the patient-derived model indicated the improvement in perfusion function after interventional surgery. With more validation by clinical cases, CMRT may be a quantitative indicator to evaluate the changes in blood perfusion after the intervention for MCA stenosis.

## Data Availability Statement

The raw data supporting the conclusions of this article will be made available by the authors, without undue reservation.

## Ethics Statement

Written informed consent was obtained from the individual(s) for the publication of any potentially identifiable images or data included in this article.

## Author Contributions

Y-HL and YC contributed conception and design of the study. YZ and RW acquired and processed clinical data. Y-HL, YC, YZ, RW, and Z-YL drafted the work or revised it critically for important intellectual content. Y-HL, YC, and Z-YL authored or reviewed drafts of the manuscript and approved the final draft. All authors contributed to the article and approved the submitted version.

## Conflict of Interest

The authors declare that the research was conducted in the absence of any commercial or financial relationships that could be construed as a potential conflict of interest.

## Publisher's Note

All claims expressed in this article are solely those of the authors and do not necessarily represent those of their affiliated organizations, or those of the publisher, the editors and the reviewers. Any product that may be evaluated in this article, or claim that may be made by its manufacturer, is not guaranteed or endorsed by the publisher.
